# miR-142-3p Expression Is Predictive for Severe Traumatic Brain Injury (TBI) in Trauma Patients

**DOI:** 10.3390/ijms21155381

**Published:** 2020-07-29

**Authors:** Cora Rebecca Schindler, Mathias Woschek, Jan Tilmann Vollrath, Kerstin Kontradowitz, Thomas Lustenberger, Philipp Störmann, Ingo Marzi, Dirk Henrich

**Affiliations:** 1 Department of Trauma-, Hand and Reconstructive Surgery, University Hospital Frankfurt, 60596 Frankfurt, Germany; mathias.woschek@kgu.de (M.W.); tilmann.vollrath@kgu.de (J.T.V.); kerstin.kontradowitz@kgu.de (K.K.); thomas.lustenberger@ksa.ch (T.L.); philipp.stoermann@kgu.de (P.S.); marzi@trauma.uni-frankfurt.de (I.M.); d.henrich@trauma.uni-frankfurt.de (D.H.); 2 Department of Traumatology, Cantonal Hospital Aarau, Tellstrasse, 5001 Aarau, Switzerland

**Keywords:** miR-142-3p, miRNA, serum, plasma, SNORD95, traumatic brain injury (TBI), polytrauma, Injury Severity Score (ISS)

## Abstract

Background: Predictive biomarkers in biofluids are the most commonly used diagnostic method, but established markers in trauma diagnostics lack accuracy. This study investigates promising microRNAs (miRNA) released from affected tissue after severe trauma that have predictive values for the effects of the injury. Methods: A retrospective analysis of prospectively collected data and blood samples of *n* = 33 trauma patients (ISS ≥ 16) is provided. Levels of miR-9-5p, -124-3p, -142-3p, -219a-5p, -338-3p and -423-3p in severely injured patients (PT) without traumatic brain injury (TBI) or with severe TBI (PT + TBI) and patients with isolated TBI (isTBI) were measured within 6 h after trauma. Results: The highest miR-423-3p expression was detected in patients with severe isTBI, followed by patients with PT + TBI, and lowest levels were found in PT patients without TBI (2^−∆∆Ct^, *p* = 0.009). A positive correlation between miR-423-3p level and increasing AIS_head_ (*p* = 0.001) and risk of mortality (RISC II, *p* = 0.062) in trauma patients (*n* = 33) was found. ROC analysis of miR-423-3p levels revealed them as statistically significant to predict the severity of brain injury in trauma patients (*p* = 0.006). miR-124-3p was only found in patients with severe TBI, miR-338-3p was shown in all trauma groups. miR-9-5p, miR-142-3p and miR-219a-5p could not be detected in any of the four groups. Conclusion: miR-423-3p expression is significantly elevated after isolated traumatic brain injury and predictable for severe TBI in the first hours after trauma. miR-423-3p could represent a promising new biomarker to identify severe isolated TBI.

## 1. Introduction

Polytrauma (PT) and severe traumatic brain injury (TBI) caused by road traffic accidents and falls are the main causes of death and disability in young patients under 45 years with immense socioeconomic impact through loss of productivity and medical and rehabilitation costs [1,2]. The treatment of patients with multiple organ injuries is challenging due to different injury patterns and severity, but also due to the complex immune response, reperfusion syndrome or endothelial barrier dysfunction. Post-traumatic overshooting immunomodulation often leads to postinjury complications, multiple organ failure (MOF) or death and is a predictor of mortality in trauma [3,4,5,6]. The brain is vulnerable to damage and failure due to its high metabolic rate and limited intrinsic energy reserve. The (neuro-)inflammatory environment subsequently leads to cell death and neurodegeneration in secondary brain damage but also to neuroreparative mechanisms at later stages. Secondary brain injury due to inflammatory processes is one of the main reasons for the worsening of the outcome after trauma [7,8,9]. Biomarkers are of highest clinical interest to detect the severity of injuries at an early stage, identifying their pathogenesis and preventing consecutive damage [10]. Their detection in serum after brain injury indicates a disturbed integrity of the blood–brain barrier (BBB), cerebrospinal fluid (CSF) recirculation into the blood via venous drainage and circulation through the lymphatic system [11,12]. The currently available biomarkers in trauma diagnostics are mostly protein-based and often lack the desired accuracy. A promising alternative could be specific microRNAs (miRNAs) released by the affected tissues. miRNAs are small non-coding RNAs with an average of 22 nucleotides. miRNA genes are transcribed by RNA polymerase II in the cell nucleus as primary miRNAs and are processed into precursor miRNAs and mature miRNAs [13,14]. The interaction of miRNAs with their target genes is dynamic and depends on many factors, such as their subcellular localization and the frequency and affinity of miRNAs and target mRNAs and their interaction. Often, miRNAs are located in specific tissues [15], depending on their function and target sequences, and therefore their potential as biomarkers for pathomechanisms is worth investigating. Injured cells can release miRNAs into the extracellular space, where they overcome endothelial barriers due to their small size and exist in stable form in the blood, facilitating peripheral sampling [16,17,18]. The identification and analysis of released miRNA patterns could provide a powerful tool to assess the outcome of patients after traumatic injury.

The main objective of the present study was to investigate the expression of specific miRNAs and their predictive value in the blood of patients (*n* = 33) within 6 h after severe trauma. In a pilot study we obtained serum and corresponding plasma samples from *n* = 20 donors, including healthy volunteers and trauma patients, to investigate the possible effects of different types of blood sample preparation on miRNA measurement. Furthermore, the total study cohort (*n* = 38) was analyzed to identify the most appropriate reference genes for the relative quantification of miRNA in trauma patients.

## 2. Results

### 2.1. Pilot Study 1: Analyzing miRNA Concentrations in Serum and Plasma Samples of Trauma Patients

**Figure 1:**
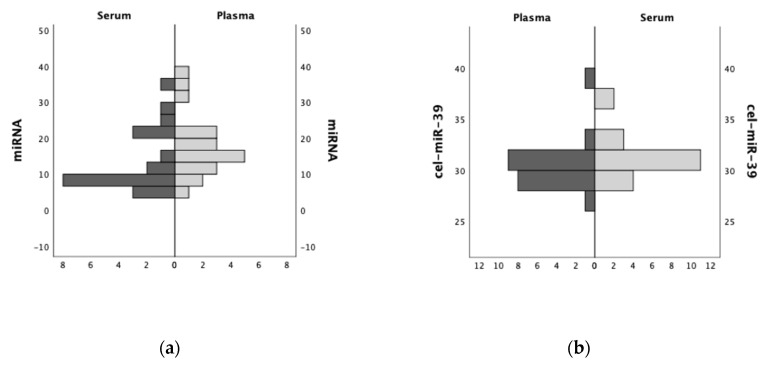
(**a**) Comparing total miRNA (ng/µL) concentrations and (**b**) exogenous spike-in control cel-miR-39 (Ct values) in serum and plasma samples (ranking distribution) from the same individuals.

Figure 1 displays the quantitative analysis and ranking distribution of miRNA and cDNA concentrations in a comparison of human serum and plasma samples.

In this pilot study, we focused on the effects of different sample types on the spectrum of circulating miRNA in the blood. The photometric determination of the miRNA concentrations was performed immediately after their isolation. Using *n* = 20 serum and *n* = 20 corresponding plasma samples from the same individuals, no significant difference in containing miRNA concentrations (serum 14.0 ± 8.4 ng/µL vs. plasma 18.0 ± 8.6 ng/µL; *p* = 0.139) was observed. Sufficient RNA isolation was verified by exogenous spike-in control cel-miR-39 via rtPCR. In addition, no significant difference in plasma and serum miRNA concentration was observed, neither by sex (*p* = 0.072) nor by age (<50 vs. ≥50 years, *p* = 0.186) of the donor.

### 2.2. Pilot Study 2: Identification of a Suitable Housekeeping miRNA in Trauma Patients

**Figure 2:**
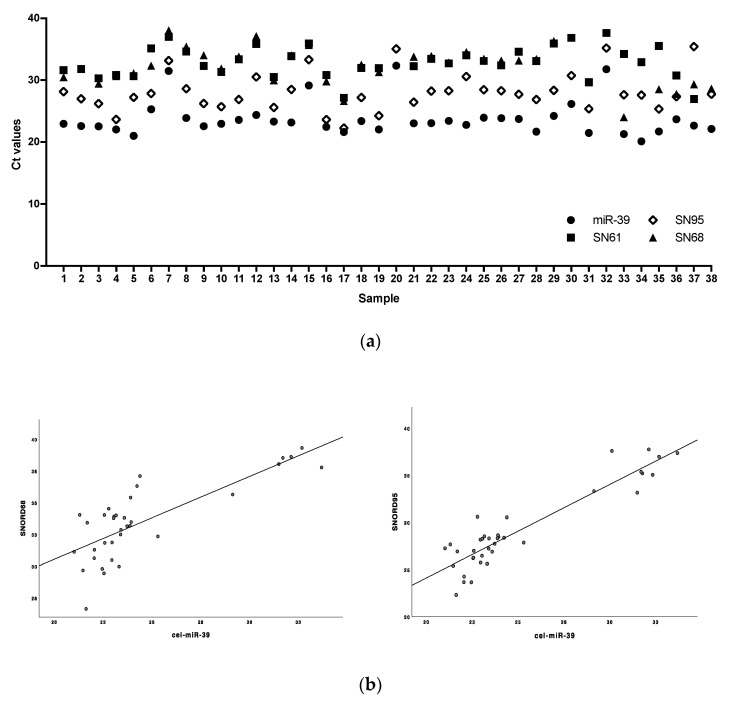
(**a**) Identification of a suitable housekeeping RNA by stability and levels of housekeeping snoRNA (Ct values of SNORD61, SNORD68, SNORD95) and (**b**) correlation of SNORD68 (left) and SNORD95 (right) with exogenous spike-in control (Ct values of cel-miR-39).

For the relative quantification of the target miRNA, we first investigated the concentration of three potential endogenous housekeeping small nucleolar RNAs (snoRNAs), SNORD61, SNORD68 and SNORD95 (Figure 2), in *n* = 38 trauma patients and healthy volunteers. The exogenous spike-in control cel-miR-39 was measured to account for differences in the efficiency of RNA extraction and Reverse Transcription (RT). The SNORD concentration was set in relation to the exogenous control cel-miR-39, which was assumed to be a constant basis by external addition during RNA isolation.

It was found that SNORD95 was stably present in all *n* = 38 serum samples. Both SNORD61 and SNORD68 were detected in *n* = 36 samples. The Ct value and normalized ∆Ct value (∆Ct = Ct_cel-miR-39_ − Ct_SNORD_) were significantly lowest for SNORD95 (∆Ct 4.3 ± 2.1) compared to SNORD61 (∆Ct 10.0 ± 5.1, *p* < 0.001) and SNORD68 (∆Ct 8.7 ± 2.4, *p* < 0.001). For SNORD61 (Pearson correlation analysis, *r* = 0.632, *p* < 0.001) and SNORD95 (*r* = 0.745, *p* < 0.001), significant positive correlations with cel-miR-39 were found. Linear regression analysis showed a higher positive correlation between cel-miR-39 and SNORD95 (*r*^2^ = 0.56, *p* < 0.001) than SNORD61 (*r*^2^ = 0.40, *p* < 0.001). Consequently, SNORD95 was selected to normalize the miRNA of the target gene for relative quantification analysis.

### 2.3. Demographic and Injury Characteristics

Table 1 displays the demographic and clinical characteristics of the trauma patients stratified by injury pattern. Of these, 8 patients were severely injured without TBI (PT), 13 patients with PT + TBI and 12 patients suffered from isTBI. 94% of these trauma patients were men. Patients with isTBI were significantly older (71 (55–77) years, *p* = 0.021) than the patients in other groups. Median (IQR) Injury Severity Score (ISS) of patients with isTBI was significantly lower (25 (21–25), *p* = 0.005), but no significant difference was found in New Injury Severity Score (NISS; *p* = 0.747). The Revised Injury Severity Classification Score (RISC II) was significantly lower in the PT cohort (*p* = 0.003). The value of Glasgow Coma Scale (GCS) of patients with PT + TBI and isTBI was significantly lower (*p* = 0.002) than after PT. The mortality in the PT cohort was 38%, 69% in the PT + TBI group and 68% died after isTBI.

### 2.4. Detection Profile of miRNAs in Trauma Patients Stratified by Injury Pattern

**Figure 3:**
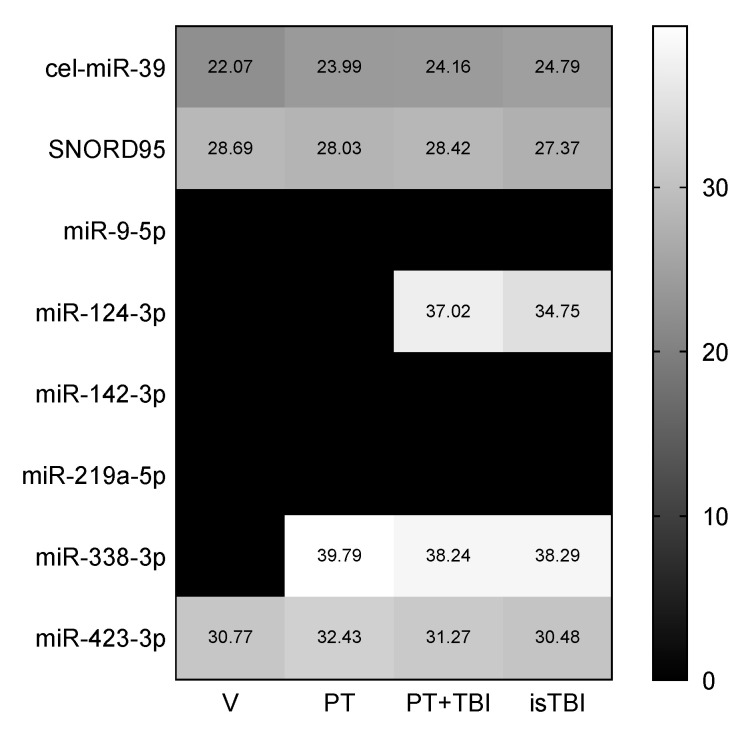
Detection profile of exogenous control cel-miR-39, housekeeping gene SNORD95 and target genes hs-miR-9-5p, -124-3p, -142-3p, -219a-5p, -338-3p and -423-3p stratified by injury pattern.

The heat map (Figure 3) shows the detection profile of the exogenous (cel-miR-39) and endogenous control (SNORD95), and of the target genes hs-miR-9-5p, -124-3p, -142-3p, -219a-5p, -338-3p and -423-3p.

Isolation was proved by the spike-in control (cel-miR-39). SNORD95 was detectable in all samples (*n* = 38). miR-423-3p expression was found in all cohorts. miR-338-3p was only detectable in trauma patients, not in healthy volunteers. miR-124-3p was only found in patients with traumatic brain injury (PT + TBI and isTBI). miR-9-5p, mirR-142-3p and miR-219a-5p could not be detected in any of the four groups.

### 2.5. Quantitative Analysis of miR-124-3p, miR-338-3p and miR-423-3p Expression in Trauma Patients

The target miRNA values were quantified relative to SNORD95 using the delta-delta Ct method (2^−∆∆Ct^) as described in Materials and Methods. Only serum miRNA levels that showed a ≥ 2.0-fold change in expression were considered positive compared to the highest level from the healthy control group. Table 2 displays ∆Ct, ∆∆Ct and 2^−∆∆Ct^ values calculated for the detected miRNA-124-3p, miR-338-3p and miR-423-3p in trauma patients stratified by injury pattern. The highest miRNA-423-3p expression was detected in patients with isTBI, followed by patients with PT + TBI; lowest miR-423-3p levels were found in the PT cohort (2^−∆∆Ct^ isTBI 1.5 ± 2.3 > PT + TBI 0.4 ± 0.2 > PT 0.2 ± 0.2).

miR-124-3p was only detected in patients with severe brain injury (PT + TBI, *n* = 7 and isTBI, *n* = 4). The 2^−∆∆Ct^ values were 0, i.e., no relevant double-fold change could be measured. The same result was found for miR-338-3p which could be detected in all trauma groups (PT *n* = 1, PT + TBI *n* = 3, isTBI *n* = 5) but not in all samples of each group.

### 2.6. Relation and Predictive Power of miR-423-3p in Severity of Injury

**Figure 4:**
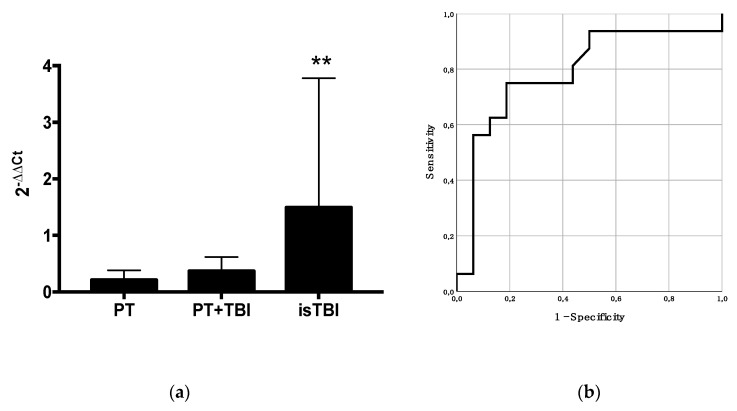
(**a**) Quantitative analysis of miR-423-3p stratified by injury pattern (*p*-value < 0.01 = **) and (**b**) predictive power of miR-423-3p for severity of brain injury (AIS_head_) in trauma patients (AUC: 0.79, *p* = 0.006, 95% CI: 0.62–0.96).

Figure 4 shows the miR-423-3p expression difference stratified by injury pattern and its predictive power according to TBI. A statistically significantly higher miR-423-3p expression in patients with isTBI (*p* = 0.009) when stratified by injury pattern was found.

Positive correlation was shown between miR-423-3p level and increasing AIS_head_ score (Spearman’s rank correlation, *r* = 0.5, *p* = 0.001), and risk of mortality (RISC II *r* = 0.3, *p* = 0.062) in all trauma patients (*n* = 33). GCS and increasing miR-423-3p concentration showed a negative correlation without statistical significance (*r* = −0.3, *p* = 0.176). In addition, a positive correlation between RISC II and severity of head injury (*r* = 0.6, *p* < 0.001) could be assessed.

The ROC analysis of miR-423-3p levels revealed a statistically significant area under the curve (AUC) to predict the severity of brain injury (AIS_head_) in trauma patients (AUC: 0.79, *p* = 0.006, 95% CI: 0.62–0.96).

### 2.7. miR-423-3p Pathway Analysis

The identification of putative target genes of miR-423-3p was performed using *miRDB*, an online database for miRNA target prediction and functional annotations. It was possible to identify 29 genes that could interact with miR-423-3p (Table 3). The genes code for proteins that cover a wide range of functions including surface receptors (FGFR2, ITGA11), cell morphogenesis (FRY) and cell fate (CBX7), but an accumulation of proteins involved in transcriptional regulation (PAPBC1, −3, CREM, BCORL1, ESRA) and intracellular signaling (RAP2C, RAB14, RAC1, CRK, PLCH1, DKK3) is evident.

## 3. Discussion

miRNAs are non-coding RNAs that affect post-transcriptional gene expression in multicellular organisms. miRNAs are transcribed by RNA polymerase II as part of capped and polyadenylated primary transcripts. This is cleaved by the Drosha ribonuclease III enzyme to produce a stem loop precursor miRNA, which is further cleaved by the cytoplasmic Dicer ribonuclease to produce mature miRNA and antisense miRNA (miRNA*). The mature miRNA is incorporated into an RNA-induced silencing complex (RISC), which in most cases leads to translation inhibition or destabilization of the target miRNA. miRNAs are involved in numerous biological processes, including differentiation and proliferation, metabolism, hemostasis, apoptosis or inflammation, and the pathophysiology of many diseases. Numerous studies have suggested circulating miRNAs as promising diagnostic and prognostic biomarkers for various diseases [18].

Serum and plasma prepared from peripheral blood is easily accessible. Although the choice between serum or plasma samples is rarely explained in publications. Previous reports on the export of miRNAs from cells have raised the question that plasma and serum may have certain differences in their miRNA content and concentrates [19,20]. Fibrinogen in plasma samples can be a source of contamination that affects extraction quality. On the other hand, the process of clotting in serum samples can alter the actual result of circulating miRNAs and increases variability [21]. Differences in peripheral blood processing could lead to significant discrepancies between studies and make it difficult to identify reliable and relevant miRNA markers [19,22,23]. In our pilot study, which compared the miRNA concentration of corresponding serum and plasma samples, no significant difference between the two biofluids was found.

The reliable assessment of the miRNA expression profile by PCR also depends on the correct data normalization by suitable reference RNA. Despite the increasing number of studies evaluating miRNAs, no consensus could be reached on the best reference, especially for miRNAs from trauma patients [24,25,26]. It is therefore of utmost importance to identify the best biofluid fraction to facilitate the discovery of miRNA-based biomarkers and to increase consistency between studies by using common reference RNA. small nuclear RNAs are widely used as an endogenous reference [19]. In our pilot study, SNORD95 (C/D Box 95) proved to be the most stable and reliable reference RNA among the snoRNAs investigated. It was found consistently in all samples and showed the highest concentration compared to SNORD61 and SNORD65 as well as a significant correlation with the exogenous isolation control cel-miR-39. To our knowledge, there are only a few studies that have investigated the expression of SNORD95, and it may be a sufficient reference RNA for miRNA analysis in trauma patients [27,28].

Of 33 trauma patients included, 94% were male and the cohort with isTBI was significantly older than the PT and PT + TBI group. In Western countries, polytrauma is the leading cause of death for people up to 45 years of age, in a male-to-female ratio of 2.6:1. Older patients cause most TBI-related hospital admissions and deaths [1,29]. In the pilot study, comparing the miRNA concentration by age and sex of donors did not result in a difference in gene expression.

In this study, the use of specific miRNAs as biomarkers was shown to have predictable value in the assessment of TBI in patients with multiple injuries [30]. Our study provides a description of changes that occur in miRNA expression during the early post-injury phase after trauma. According to the “human miRNA tissue atlas” a ubiquitous miR-423-3p expression was detected across human tissue [15]. In this study, miR-423-3p was detected in all samples including healthy volunteers but was significantly higher expressed after isTBI than after PT or PT + TBI. In addition, a significant correlation was shown between the severity of injury (ISS) and level of consciousness (GCS) and the relevant predictive value for severity of brain injury (AIS_head_). Changes in miR-423-3p expression after CNS injury and spinal cord injury in the early phase after injury have already been shown in porcine serum [31]. Furthermore, different TBI intensities were shown to induce a differentiated miRNA expression profile in the brain after injury. Among other markers, miR-423 was identified, which may be involved in the pathophysiology of mild TBI [32]. miR-423-3p and miR-142-3p were identified as markers after traumatic brain injury that could be useful for distinguishing severity and improvement over time [33]. miR-142-3p could not be detected in our study. The miR-423 gene is located in a frequently amplified region of chromosome 17q11.2 and can produce two mature sequences: miR-423-3p and miR-423-5p [34]. It has already been reported that miR-423-3p acts like an oncogene by promoting cell proliferation, cell cycle progression, clonogenicity, cell migration and invasion. miR-423-3p plays a predominant role in oncogenic autophagy via Bcl-2 interacting mediators of the cell death (bim) axis and in the anti-apoptotic p-Akt and p-ERK1/2 signaling axis [35]. In our study, it was possible to identify 29 genes that could interact with mirR-423-3p. The genes code for proteins that cover a wide range of functions including surface receptors (FGFR2, ITGA11), cell morphogenesis (FRY) and cell fate (CBX7). But an accumulation of proteins involved in transcriptional regulation (PAPBC1,-3, CREM, BCORL1, ESRA) and intracellular signaling (RAP2C, RAB14, RAC1, CRK, PLCH1, DKK3) is evident. MiR-423-3p appears to regulate a variety of cellular processes. Looking at the target genes, most of them are involved in cellular activation (signal transduction, transcription) and thus in all possible processes after trauma (regeneration, inflammation, etc.). Interestingly, miR-423-3p was significantly higher concentrated in serum/plasma after isTBI compared to PT, and especially PT + TBI. In previous studies, we have already seen that the PT + TBI cohort behaves unexpectedly differently to the isTBI group with respect to neuromarker expression, e.g., the serum level of neuron-specific enolase 2 (NSE) is lower after PT + TBI than after isTBI. At the same time, it could be shown that TBI has a relevant influence on the immune response (e.g., IL-6 and IL-10) after trauma, which can result in altered barrier conditions and local acute inflammation and subsequent tissue damage. We have shown that IL-10 is secreted significantly higher after PT + TBI than after isTBI [9,36]. IL-10 is an anti-inflammatory cytokine that regulates and limits acute inflammation in response to trauma to prevent tissue damage, such as secondary brain damage following TBI [37,38]. In contrast, the pro-inflammatory cytokine IL-6 modulates the expression of tight junction proteins in cerebral microvasculature, and the release of adhesion molecules in plasma of polytrauma patients (ISS ≥18) correlates with organ dysfunction [39]. Our hypothesis for the lower miR-423-3p level in the PT + TBI group is an immunomodulatory effect of TBI in polytrauma. Similarly, more dominant trauma damage outside the CNS during polytrauma may alter the serological composition to mask the increase in miRNA-423-3p. An important pathophysiological factor in the development of posttraumatic complications is the dysfunction of the external (skin) and internal paracellular blood and organ barriers, including the brain (BBB) [40]. miRNAs themselves are involved in maintaining the integrity of the BBB by targeting the 3′-UTR of claudin-1, JAM 3, occlusive and tight junction-associated protein 1 [41]. Inflammation modulates the expression of tight junction proteins in the cerebral microvasculature of sheep, and the release of adhesion molecules in plasma of polytrauma patients (ISS ≥ 18) correlates with disease severity and organ dysfunction [39]. Due to the increased permeability of the organ barriers, both metabolites and larger molecules such as small peptides (e.g., S100b) and small polynucleotides can enter the bloodstream and be detected as specific biomarkers [12]. Identifying the severity of injuries at an early stage and predicting their development in order to prevent secondary damage are of paramount interest in trauma treatment, with the detection of biomarkers playing an important role [42]. It can be assumed that brain-specific miRNAs are released from broken cells, just like the proteins of known neuromarkers.

With regard to altered gene expression after brain injury, miR124-3p is one of the most frequently investigated miRNAs. miR-124-3p is specifically expressed in the CNS [15]. In our study, miR-124-3p was only detected in patients with (concomitant) TBI (PT + TBI and isTBI). Increased miR-124-3p in microglial exosomes following traumatic brain injury inhibits neuronal inflammation and contributes to neurite outgrowth [43,44]. Other studies found that miR-124-3p is a modulator of molecular networks relevant for hippocampal pathologies following injury in experimental models and in humans [45]. The concentration of miR-124-3p can be used as a predictor for neurologic outcome and survival after cardiac arrest [46]. Hamzei et al. found that miR-124 overexpression in mice induced early focal ischemia which reduced the number of microglia/macrophages, leading to neuroprotective and anti-inflammatory effects [47]. Although no relevant double-fold change could be measured after semiquantitative analysis (2^−∆∆Ct^), in this study miR-124-3p was not detectable in healthy volunteers and the PT cohort, so that a specific expression/release after TBI can be assumed.

A slightly increased serum concentration of miR-338-3p after trauma, especially after PT + TBI and isTBI, was found, not in healthy volunteers. Although miR-338-3p is predominantly expressed in CNS, it could also be detected in peripheral nerves [31], which might explain an unspecific detection in one PT patient due to a larger peripheral nerve damage. The data on miR-338-3p, especially after trauma, are very thin. More studies have been done on brain tumors in which low expression of miR-338-3p is associated with increased mortality and disease progression by PTEN/Akt pathway regulation in patients with brain tumors [48,49]. Studies indicate that prevention of traumatic brain injury-induced ROS production decreases BBB disruption, neuronal death and microglial activation, which may have high therapeutic potential to reduce traumatic brain injury-induced neuronal death [13]. In addition, a number of studies have demonstrated an antioxidant role for tumor-suppressor proteins, activating the expression of antioxidant genes in response to oxidative stress. Tumor-suppressor genes regulate diverse cellular activities, including DNA damage repair, cell cycle arrest, cell proliferation, cell differentiation, migration and apoptosis. It has been demonstrated that upregulation of PTEN causes modulation of PI3K/Akt signaling to reduce ROS generation in cells [50]. There is possibly a post-traumatic activation of the PI3K/AKT pathways, which is regulated by a miR-338-3p expression.

In this study, both miR-219a-5p and miR-9-5p could not be detected in serum samples. miR-9 was shown to be regulated in brain tissue after experimental TBI and detected in CSF [51]. It has been shown that miR-219a-5p induces a change in the expression of cleaved caspase-3 in the post-TBI model of rat cortical neuronal cells, thereby inducing neuronal apoptosis [52]. It is possible that in these trauma patients, miR-219a-5p and miR-9 did not enter the venous bloodstream in relevant amounts and therefore could not be detected in our serum samples. Both miRNAs are considered to be human brain-specific, but especially for miR-219a-5p, it has already been shown that it is not stably expressed [15]. Another study showed that miR-9 is highly enriched in developing nervous systems of vertebrates and its expression is preferably associated with neurogenic precursors [53]. Possibly an increase in miR-9-5p could be detected with the onset of the resorptive phase and neuroreparative mechanisms. In patients with rheumatoid arthritis, miR-9-5p is downregulated by IL-6 and TNF-α and has a preventive effect on the development of peripheral neuropathy [54]. Perhaps the miR-9-5p expression is more dependent on the inflammatory response that sets in after severe trauma.

### Limitations of the Study

The main limitation is the retrospective nature of the data analysis; however, all clinical data were collected prospectively, and only the miRNA was measured later on.

Furthermore, the patients with isTBI were significantly older than the comparison groups. Finally, no conclusions can be drawn as to how age may have altered the miRNA expression―although there is no evidence that this might have an effect. It is in the nature of trauma that the patient cohort with isTBI is significantly older, which is due to the most common trauma mechanism in older people (fall from a low height) and possibly pre-medication with oral anticoagulation. Patients with previously known neurodegenerative diseases (Alzheimer’s, Parkinson’s, etc.) and organic brain syndromes (epilepsy, schizophrenia, etc.) were excluded. However, due to the retrospective nature of the study and the relevant age difference between the cohorts, it is problematic to completely exclude most (neurodegenerative) diseases. A further advantage of future prospective studies could be to optimize the exclusion of relevant diseases in order to further reduce possible co-founding effects.

Another important point of confounding factors is the presence of alcohol or drugs at the time of accident. Blood alcohol levels are routinely determined at the time of admission via our emergency room. Positive results were only found in the isTBI cohort. The comparison of the average value of miR-423-3p (2^−∆∆Ct^) of the alcohol-positive group (ethanol+) with the group without a positive blood alcohol level (ethanol-), showed no significant difference (2^−∆∆ Ct^ ethanol + vs. 2^−∆∆ Ct^ ethanol-: *p* = 0.788)—with critical consideration of the small number of cases. Two subjects in the PT + TBI group tested positive for benzodiazepines due to preclinical sedation. There were no abnormalities in the miR423-3p level of these patients. Especially chronic consumption can have a relevant influence on brain organic structures, cell function and miRNA expression.

The variance in the individual values is large, especially for miRNA, which is due to potential confounders such as volume administration, blood products and shock, which are difficult to standardize. For subsequent studies, further measurements and later points in time should be considered to show the possible kinetics in gene expression.

## 4. Materials and Methods

This retrospective study was performed at the University Hospital Frankfurt, Goethe University after approval by the Institutional Review Board (89/19) in accordance with the Declaration of Helsinki and following STROBE guidelines [55]. Written informed consent was obtained for enrolled patients and volunteers or their legally authorized representatives. A retrospective analysis of prospectively collected data in severely injured trauma patients (ISS ≥ 16) is provided. The study cohort was retrospectively divided into multiple injured patients without TBI ([PT], AIS_head_ ≤ 1) or with severe TBI ([PT + TBI], AIS_head_ ≥ 4; AIS of other body area ≥ 3) and patients with isolated TBI ([isTBI], AIS_head_ ≥ 4, all other AIS ≤ 1). Exclusion criteria were previously known chronic, systemic inflammatory or metabolic syndrome, polyneuropathy, critical illness syndrome, neuro-degenerative diseases (Alzheimer, Parkinson, etc.), organic brain syndromes (epilepsy, schizophrenia, etc.), stroke, minor age < 18 years and sepsis. Healthy volunteers (V) served as the control group.

Blood samples (*n* = 38) were collected of patients admitted to the emergency room of our level-1 trauma center located in the University Hospital of the Goethe University; Frankfurt am Main within 6 h after trauma. The blood was collected in 7.5 mL/2.7 mL tubes (S-Monovette^©^, Sarstedt Inc., Nümbrecht, NRW, Germany) containing 1.6 mg EDTA K (plasma samples) or silicate coated granules and polyacrylester gel (serum samples). Within 60 min, samples were centrifuged for 15 min at 3500 rpm and 4 °C. The upper plasma/serum phase was transferred and stored at −80 °C until assayed.

Isolation of miRNA was performed with 200 µL of human plasma or serum using the miRNeasy serum/plasma kit (Qiagen Inc., Hilden, NRW, Germany). For optimized lysis conditions to purify miRNA, we used a qiazol separation kit (Qiagen Inc., Hilden, NRW, Germany). For quality control of the RNA isolation and cDNA synthesis steps, spike-in control cel-miR-39 (miRNA spike-In Kit, for RT, Qiagen Inc., Hilden, NRW, Germany) was added. Concentration of isolated RNA was determined photometrically using a spectrophotometer (Nanovue^©^, Harvard Bioscience Inc., Holliston, MA, USA) at a path length of 500 nm. Reverse transcription (RT) was performed with 20 ng miRNA using miScript II RT kit (Qiagen Inc., Hilden, NRW, Germany). PCR arrays (400 ng cDNA) were done according to manufacture instructions using miScript SYBR^®^ Green PCR Kit (Qiagen Inc., Hilden, NRW, Germany). Real-time PCR (rtPCR) was performed using Stratagene Mx3005P QPCR Systems (Agilent Technologies Deutschland, Waldbronn, BW, Germany) with thermal profile of 1 cycle with 15 min of 95 °C, 40 cycles with 15 sec of 94 °C, 30 sec of 55 °C and 30 sec of 70 °C followed by dissociation curve. The array contained assays for miRNAs of interest (hsa-miR-9-5p, -124-3p, -142-3p, -219a-5p, -338-3p, -423-3p; hsa (*homo sapiens*)), endogenous reference snoRNAs (hsa-SNORD61, -SNORD68 and -SNORD95) and exogenous control cel-miR-39.

Identification of putative miR-423-3p target genes was performed using the *miRDB* database (http://mirdb.org/, accessed on 22/07/2020). *miRDB* is an online database for miRNA target prediction and functional annotations [56]. All information on the putative protein functions was taken from the NCBI gene bank (https://www.ncbi.nlm.nih.gov/gene, accessed on 22/07/2020).

### Statistical Analysis

Continuous normally distributed variables were summarized using means ± standard deviation (*SD*), while categorical or continuous variables with skewed distributions were summarized using medians with interquartile ranges (*IQR*). The selection of the best housekeeping miRNA for the study with trauma patients was based on the expression of the three SNORDs compared to the exogenous spike-in control added during isolation of miRNA. Using always the same amount of miRNA and cDNA, a constant miR-39 level was assumed and the SNORD expression pattern was compared and correlated with that of cel-miR-39. The *p*-values for categorical variables were derived from the two-sided Fisher’s exact test, and for continuous variables from the Mann–Whitney U test or the Kruskal–Wallis test. Significant values were adjusted by the Bonferroni post hoc test. Pearson’s and Spearman’s rank correlation coefficients were calculated to determine correlations between miRNA and injury characteristics. A *p*-value <0.05 was considered to be statistically significant.

For relative quantitative analysis of target miRNA, the delta-delta Ct method (2^−∆∆Ct^) was used according to Livak et al. [57]. After normalization of Ct values of the target genes (all samples, *n* = 38) against the housekeeping gene (∆Ct_Target_ = Ct_SNORD95_ − Ct_Target_), the ∆∆Ct value was calculated using healthy individuals as the control cohort (∆∆Ct_Target_ = ∆Ct_[V]_ − ∆Ct_Target_):

(1). ∆Ct_Target_ = Ct_SNORD95_ – Ct_Target_;
(2). ∆∆Ct_Target_ = ∆Ct_[V]_ – ∆Ct_Target_;
(3). 2^−∆∆Ct^.

All analyses were performed using the Statistical Package for Social Sciences (SPSS for Mac^©^), version 26 (SPSS Inc., Chicago, IL, USA). The graphics were created using GraphPad Prism 7 for Mac^©^ (GraphPad Software Inc., San Diego, CA, USA).

## 5. Conclusions

miR-423-3p expression is significantly elevated after isTBI and predictable for severe traumatic brain injury in the first hours after trauma. In addition, significant correlations were shown between miR-423-3p levels and injury severity and impairment of consciousness after TBI. miR-423-3p could represent a promising new biomarker to identify severe isTBI. Another possible application could be in the area of risk groups, such as sports with a high risk of head injuries, such as football and boxing, or blast injuries during military service, in order to better assess the individual risk of brain damage to the injured person. Furthermore, it could be shown that there is no relevant difference in RNA concentration in serum or plasma samples and SNORD95 is a suitable reference RNA for miRNA expression studies in trauma patients.

## Figures and Tables

**Table 1:** Demographic and injury characteristics.

	PT (*n* = 8)	PT + TBI (*n* = 13)	isTBI (*n* = 12)
	Median (IQR)	Median (IQR)	Median (IQR)
Age (y)	52 (42–66)	48 (32–61)	71 (55–77)
ISS (pts)	37 (24–43)	34 (32–42)	25 (21–25)
NISS (pts)	47 (31–50)	48 (39–57)	45 (37–62)
RISC II (%)	4 (2–11)	39 (14–60)	33 (17–83)
AIS_head_ (pts)	0	5 (4–5)	5 (4–5)
AIS_thorax_ (pts)	4 (3–4)	3 (3–4)	0
AIS_abdominal_ (pts)	3 (2–4)	0 (0–1)	0
AIS_extremities_ (pts)	4 (2–5)	0 (0–3)	0
GCS (pts)	15 (11–15)	3 (3–8)	3 (3–12)

Abbreviations: PT = polytrauma, isTBI = isolated traumatic brain injury, IQR = Interquartile range, y = years, ISS = Injury Severity Score, pts = points, NISS = New Injury Severity Score, RISC II = Revised Injury Severity Classification, Version 2, AIS = Abbreviated Injury Scale, GCS = Glasgow Coma Scale.

**Table 2:** Quantitative analysis of miR expression in trauma patients stratified by injury pattern.

		miR-124-3p		miR-338-3p		miR-423-3p	
		*n*	mean ± SD	*n*	mean ± SD	*n*	mean ± SD
PT	Ct			1	39.8 ± 0	8	32.4 ± 2.9
	∆Ct				13.6 ± 0		4.7 ± 1.2
	∆∆Ct				13.6 ± 0		2.6 ± 1.2
	2^−∆∆Ct^				0.0		0.2 ± 0.2
PT + TBI	Ct	7	37.0 ± 3.0	3	38.2 ± 1.3	13	31.3 ± 3.5
	∆Ct		11.8 ± 3.8		13.5 ± 1.9		3.9 ± 1.3
	∆∆Ct		11.8 ± 3.8		13.5 ± 1.9		1.8 ± 1.3
	2^−∆∆Ct^		0.0		0.0		0.4 ± 0.2
isTBI	Ct	4	34.7 ± 2.7	5	38.3 ± 1.6	12	30.7 ± 1.4
	∆Ct		7.5 ± 1.3		10.4 ± 2.0		2.4 ± 1.5
	∆∆Ct		7.5 ± 1.3		10.4 ± 2.0		0.3 ± 1.5
	2^−∆∆Ct^		0.0		0.0		1.5 ± 2.3

Abbreviations: PT = polytrauma, isTBI = isolated traumatic brain injury, SD = standard deviation, Ct = cycle threshold. Quantitative analysis of target miRNA delta-delta Ct method (2^−∆∆Ct^): after normalization of Ct values of the target genes (all samples, *n* = 38) against the housekeeping gene (∆Ct_Target_ = Ct_SNORD95_ − Ct_Target_), the ∆∆Ct value was calculated using healthy volunteers as the control cohort (∆∆Ct_Target_ = ∆Ct_[V]_ − ∆Ct_Target_).

**Table 3:** Pathway analysis was performed using *miRDB* database and revealed 29 potential target genes for miR-423-3p.

Target Rank	Target Score	Gene Symbol	Gene Description	Protein Function
1	96	PABPC1	poly(A) binding protein cytoplasmic 1	Binds to RNA, translation initiation
2	85	PABPC3	poly(A) binding protein cytoplasmic 3	Binds to RNA, translation initiation
3	83	RAP2C	RAP2C, member of RAS oncogene family	small GTPases that act as molecular switches to regulate cellular proliferation, differentiation, and apoptosis
4	78	FRY	FRY microtubule binding protein	Cell morphogenesis
5	77	CBX7	chromobox 7	Controls the lifespan of several normal human cells
6	77	CREM	cAMP responsive element modulator	Transcription factor that binds to the cAMP responsive element
7	76	RAB14	RAB14, member RAS oncogene family	Low-molecular mass GTPase, involved in intracellular membrane trafficking
8	74	FGFR2	fibroblast growth factor receptor 2	The extracellular portion of the protein interacts with fibroblast growth factors, ultimately influencing mitogenesis and differentiation.
9	73	BCORL1	BCL6 corepressor like 1	Can interact with several different class II histone deacetylases to repress transcription.
10	70	RAC1	Rac family small GTPase 1	GTPase which belongs to the RAS superfamily, appears to regulate a diverse array of cellular events, including the control of cell growth, cytoskeletal reorganization and the activation of protein kinases.
11	70	ESRRA	estrogen related receptor alpha	Nuclear receptor that is most closely related to the estrogen receptor, acts as a site-specific transcription factor
12	70	ITGA11	integrin subunit alpha 11	Encodes an alpha integrin.
13	69	LGALSL	galectin like	unknown
14	68	GYPC	glycophorin C (Gerbich blood group)	Plays an important role in regulating the mechanical stability of red cells.
15	66	PROZ	protein Z, vitamin K dependent plasma glycoprotein	The encoded protein plays a role in regulating blood coagulation
16	65	HS6ST2	heparan sulfate 6-O-sulfotransferase 2	Interacts with various ligands to influence cell growth, differentiation, adhesion and migration
17	65	ZNF135	zinc finger protein 135	unknown
18	64	DLL1	delta like canonical Notch ligand 1	Plays a role in mediating cell fate decisions during hematopoiesis
19	62	ZBTB46	zinc finger and BTB domain containing 46	unknown
20	60	CRK	CRK proto-oncogene, adaptor protein	Is involved in several signaling pathways, recruiting cytoplasmic proteins in the vicinity of tyrosine kinase
21	59	ACOX3	acyl-CoA oxidase 3, pristanoyl	Is involved in the desaturation of 2-methyl branched fatty acids in peroxisomes.
22	59	TRDN	triadin	Integral membrane protein that contains a single transmembrane domain
23	59	DKK3	dickkopf WNT signaling pathway inhibitor 3	Involved in embryonic development through its interactions with the Wnt signaling pathway. It may function as a tumor suppressor gene.
24	58	KLHL29	kelch like family member 29	Binding interactions with other proteins, e.g., actins
25	54	SLC11A2	solute carrier family 11 member 2	Transports divalent metals and is involved in iron absorption.
26	54	ZNF16	zinc finger protein 16	Is involved in the differentiation of erythroid and megakaryocytic cells.
27	51	PLCH1	phospholipase C eta 1	cleaves PtdIns (4,5) P2 to generate second messengers IP3 and DAG
28	50	CALML3	calmodulin like 3	unknown
29	50	GIPC3	GIPC PDZ domain containing family member 3	required for postnatal maturation of the hair bundle and long-term survival of hair cells and spiral ganglion in the ear.

## Abbreviations

AIS	Abbreviated Injury Score
ISS	Injury Severity Score
NISS	New Injury Severity Score
RISC II	Revised Injury Severity Classification Score II
PT	Polytrauma
(is)TBI	(isolated) Traumatic Brain Injury
MOF	multiple organ failure
BBB	Blood–brain barrier
SNORD	small nuclear RNAs
GCS	Glasgow Coma scale
